# Quantification of dislocation nucleation stress in TiN through high-resolution *in situ* indentation experiments and first principles calculations

**DOI:** 10.1038/srep15813

**Published:** 2015-11-05

**Authors:** N. Li, S.K. Yadav, X.-Y. Liu, J. Wang, R.G. Hoagland, N. Mara, A. Misra

**Affiliations:** 1 Materials Physics and Applications Division, MPA-CINT, Los Alamos National Laboratory, Los Alamos, New Mexico 87545, USA; 2 Materials Science and Technology Division, MST-8, Los Alamos National Laboratory, Los Alamos, New Mexico 87545, USA; 3 Department of Mechanical and Materials Engineering, University of Nebraska-Lincoln, Lincoln, NE 68583, USA; 4 Department of Materials Science and Engineering, University of Michigan, Ann Arbor, Michigan 48109, USA

## Abstract

Through *in situ* indentation of TiN in a high-resolution transmission electron microscope, the nucleation of full as well as partial dislocations has been observed from {001} and {111} surfaces, respectively. The critical elastic strains associated with the nucleation of the dislocations were analyzed from the recorded atomic displacements, and the nucleation stresses corresponding to the measured critical strains were computed using density functional theory. The resolved shear stress was estimated to be 13.8 GPa for the partial dislocation 1/6 <110> {111} and 6.7 GPa for the full dislocation ½ <110> {110}. Such an approach of quantifying nucleation stresses for defects via *in situ* high-resolution experiment coupled with density functional theory calculation may be applied to other unit processes.

Understanding the mechanics of dislocation nucleation is a fundamental challenge due to the difficulty of direct experimental measurements at the atomic scale^1^,^2^,^3^,^4^,^5^,^6^,^7^,^8^,^9^. Under athermal conditions, the resolved shear stress required for dislocation nucleation is expected to be on the order of the theoretical limit of shear strength for perfect crystals. Owing to the inherent stochastic nature of dislocation nucleation at finite temperature^10^,^11^, the stresses for dislocation nucleation are below the athermal limit and correlated with test temperature^12^,^13^,^14^, deformation procedure (strain rate^15^,^16^ and stress state^13^) and nucleation sites (such as free surface, grain/interphase boundaries^17^,^18^,^19^,^20^,^21^ or cracks^22^,^23^,^24^,^25^). Nanoindentation experiments have been employed to explore nucleation phenomena from surfaces^26^,^27^. Using nanoindentation, Angstrom-level control on the displacement and nano-Newton-level control on the load, the onset of plastic behavior is characterized as a displacement excursion/burst or “pop-in” during load-controlled indentations^28^ or sudden force drop in displacement-controlled mode^29^. The stress field beneath the indenter tip is calculated using the Hertz contact model and the maximum shear stress is treated as the critical stress for dislocation nucleation^30^,^31^. However, *ex situ* studies cannot directly bridge the load-displacement curve with unit processes under the indenter. Molecular dynamics (MD)^32^ simulations have been employed to quantitatively study mechanics of dislocation nucleation, but are limited by the availability of reliable potentials and up to 10 orders of magnitude higher strain rate in simulations as compared to experiments.

*In situ* indentation in a TEM offers an excellent tool to unveil dislocation nucleation in various environments, such as free surfaces^4^,^33^, grain boundaries^34^, internal single-ended spiral sources^1^,^35^, twin boundaries^36^,^37^, interphase boundaries^38^,^39^,^40^ and cracks^5^. From quantitative load–displacement measurements in polycrystalline Al film using *in situ* indentation in a TEM^2^,^26^,^33^, Minor *et al.* estimated critical resolved shear stress for dislocation nucleation in both dislocation-free and deformed domains and found them close to the theoretical shear strength. In this Letter, we explored the nucleation mechanics at atomic scale by performing *in situ* indentation tests in a TEM at high-resolution mode. We characterized dislocation nucleation processes and computed the critical strains according to the recorded atomic displacements. The critical stresses are calculated by using first principles density functional theory to deal with non-linear elasticity of large strains.

TiN is one of most thoroughly investigated transition metal nitrides^41^,^42^,^43^,^44^,^45^,^46^,^47^,^48^,^49^,^50^ and chosen in this study. TiN with the rock-salt (B1) crystal structure has three possible slip systems {110} <110>, {001} <110> and {111} <110>. In order to reveal the nucleation behavior of dislocations on the three slip systems, we designed our experimental setup to selectively favor one or two slip systems. Table 1 shows the calculated Schmid factors for possible slip systems for the two cases of indentation direction along <111> and <001>, respectively. The Schmid factors exhibited in Table 1 show that the systems {111} <110> and {100} <110> will be primarily activated for the indentation along <111> and the systems {110} <110> and {111} <110> will be primarily activated for the indentation along <100>. We thus deposited TiN films epitaxially on single-crystal MgO (100) & (111) at 650 °C. *In situ* nanoindentation studies were conducted at room temperature with a Nanofactory scanning tunneling microscopy (STM) platform inside a Tecnai G(2) F30 transmission electron microscope. We performed indentation tests along two crystallographic directions <111> and <100>, as shown in Fig. S1 (also see movies in Supplementary Materials), and characterized two activated slip systems {111} <110> and {110} <110>.

Figure 1a shows the HRTEM image of the TiN crystal under *in situ* straining by a tungsten indenter appearing in the lower right corner of the image (see Supplementary Movie 1). This image is one of a series of images collected at a rate of 3 frames per second during the test. A grain boundary separates the upper left grain from the lower right. The indenter is located at the surface of the upper grain away from the boundary. The loading direction is closely parallel to the 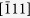 direction of the upper grain and the electron beam direction is along [110]. The region beneath the tip (marked as a square box in Fig. 1a) has been magnified in Fig. 1b. During an interval of 0.3 seconds, an offset along 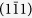 plane of 7.5 nm in magnitude is observed and is indicated by a dashed line in Fig. 1c, corresponding to the nucleation of a partial dislocation and the formation of a stacking fault. We further characterized the Burgers vector of the nucleated partial dislocation. A Burgers circuit starting at S and ending at F is shown in Fig. 1e and the Burgers vector is a_0_/6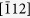 with the line sense ξ = [110]. To clearly show the offset associated with the partial dislocation during nucleation, we chose atoms in lower right corner (region I in Fig. S2) as a reference and remap atomic displacements in other regions. Supplementary Movie 2 reveals the collective shift of atoms in regions II, III and IV with respect to region I. This nucleated partial dislocation is observed for 1 second during the indentation. With continued compression, the offset region recovers (as shown in Fig. 1d), corresponding to the scenario that a trailing partial has been nucleated and combined with the leading partial to form a perfect lattice dislocation with Burgers vector ½ <110>, which has glided into the sample and is invisible in the frame in Fig. 1d.

To quantify the critical strains corresponding to the nucleation of the partial dislocation, a lattice strain analysis was performed, based on the HRTEM image (Fig. 1b) acquired 0.3 s before the nucleation. The method is developed based on the least squares determination of the strain ellipse at a lattice site in the plane of a lattice image defined relative to a perfect reference lattice (see details in Supplementary Materials). Figure 2a–c show strains *ε*_*yy*_, *ε*_*xx*_, and *ε*_*xy*_ in the nucleation region beneath the indenter, where the coordinate system is x = 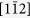 , y = 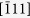 , and z = 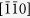. The presence of the scattered blank regions is due to image distortion under straining. The magnitude of compressive strains *ε*_yy_ decreases with distance from the indenter (Fig. 2a), roughly in agreement with Hertzian theory. Due to large strains beyond linear elasticity right beneath the indenter, stresses associated with such strains are calculated by using first principles density functional theory (DFT) (see Method for model details). Figure 2d shows the contour of the resolved shear stress on the 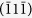 plane. Superimposing the contour on the TEM micrograph helps identify the stress gradient beneath the indenter (in Fig. 2d), consistent with the trend in compressive strain *ε*_yy_. There is a small region of large strains close to the indenter. The strain component *ε*_*xx*_ is predominantly tensile (Fig. 2b). With these data, the average strain tensor at the location of the dislocation nucleation is 
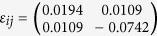
. The corresponding DFT stress is 
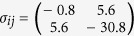
 (GPa) under the indenter, with the critical shear stress of 13.8 GPa for the nucleation of a partial dislocation on {111}.

Figure 3a shows a TEM image when the e-beam direction is along [100] and the compressive loading direction is along [001]. Nucleation and glide of a full dislocation was observed (see Supplementary Movie 3). Figures 3b,c show, in two successive frames, the trajectory of a lattice dislocation on 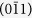 plane. The dislocation is nucleated from the free surface in contact with the indenter and glides into the sample. The Burgers vector of the nucleated dislocation has been identified to be a_0_/2[110] with the line sense ξ = 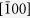. Lattice strain analysis based on HRTEM images determined the local strains under the indenter 
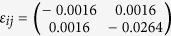
 and the corresponding DFT stresses 
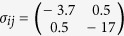
 (GPa). This corresponds to a resolved shear stress of 6.7 GPa, greater than the estimation made by Oden *et al.* based on the Hertz contact model^47^.

{110} <110> has so far been commonly believed to be the room temperature slip system in TiN while the slip system {111} <110> has never been observed experimentally. Here we have for the first time confirmed two activated slip systems, {111} <110> and {110} <110>, with respect to indentation direction along <111> and <100>, respectively. Table 2 summarized the experimental results for the two indentation orientations.

The possible reason for the higher nucleation stress for a partial dislocation on {111} plane than the full dislocation on {110} plane may be the larger Peierls stress for {111} <110>. To rationalize these observations, we calculated generalized stacking fault energies (GSFE) of {111}, {110}, and {100} planes using DFT as a function of shear displacements along both <110> and <112> directions. Figure 4a shows variation of GSFE of {110} and {100} planes with shear displacements along <110> direction. The results suggest that dislocations a ½ <110> Burgers vector are energetically favored on {110} and {001} slip planes, and {100} <110> experiences a higher unstable stacking energy of 2.5 J/m^2^. Figure 4b shows variation of GSFE of {111} plane with shear displacements along both <110> and <112> directions. The unstable stacking fault energy for a_0_/2 <110> shear is 2.5 J/m^2^. For the shear of a_0_/3 <112>, the unstable (*γ*_*usf*_)^51^ and stable stacking fault energies (*γ*_*sf*_) are 1.4 J/m^2^ and 1.1 J/m^2^ respectively. The results suggest that the partial dislocation with Burgers vector of a_0_/6 <112> prefers to nucleate and shear between two Ti layers. Following the notation of Frank and Nicholas^52^, we label planes of atoms containing Ti by Roman letters and planes of atoms containing N by Greek letters (in Fig. 4c,d). The stacking sequence of the {111} planes of TiN can be expressed as …*AγBαCβAγBαCβ*… In Fig. 4d, at displacement of a_0_/3 <112>, which corresponds to a Shockley partial vector, the stacking sequence is changed to …*AγBαCβ*|*CβAγBα*…, where “|” indicates the position of the fault plane. In this configuration, one Ti layer is displaced relative to the neighboring Ti layer in the “anti-twinning” sense. This corresponds to a high energy stacking fault structure in fcc metals. In Fig. 4b, at displacement of a_0_/6 <112>, which corresponds to the Shockley partial in the “twinning” sense, another type of stacking fault exists. However, formation of this type of stacking fault requires a cooperative motion of the interfacial nitrogen atoms within the slip plane, the so called “synchroshear mechanism”^52^, which is typically diffusion assisted. Since our experiments were not conducted at high temperatures, this type of stacking fault is not observed.

In summary, *in situ* indentation in a TEM under high-resolution coupled with image analysis has been successfully used to observe and quantify dislocation nucleation in TiN. For two different indentation orientations, <111> and <001>, we identified two slip systems in terms of dislocation characters and corresponding critical nucleation strains. Critical shear stresses were obtained using density functional theory to deal with non-linear elastic behavior of the high critical strains, ~13.8 GPa for nucleating a partial dislocation on {111} and ~6.7 GPa for nucleating a full dislocation on {110}. We believe that such approach can be applied to other unit phenomena, such as dislocation nucleation from boundaries or cracks, dislocation multiplication or interaction, *etc*.

## Methods

### *In situ* compression experiments

Our experiments were conducted inside a FEI Tecnai F30 field emission gun transmission electron microscope (TEM) equipped with a Nanofactory TEM-STM system. The TEM was operated at 300 kV, with a point-to-point resolution around 0.2 nm. The videos were recorded by a CCD (charge-coupled device) camera at 3 frames per second. TiN TEM foils were attached on a piezo-operated scanning tunneling microscope (STM) probe with silver paste. An etched W wire, with tip radius less than 100 nm, has been used as the indentation tool. And the compressive displacement rate is well controlled to be ~0.1 nm/s.

### DFT simulations

The DFT calculations were performed using the *Vienna Ab initio Simulation Package* (VASP), employing the Perdew, Burke and Ernzerhof (PBE) exchange correlation functional and the projector-augmented wave (PAW) methodology. The calculated lattice parameters, bulk modulus, and elastic constants of TiN in the rock salt crystal structure were in excellent agreement with other DFT calculations and experimental values^44^,^45^,^52^. For the dislocation structure calculations, a single partial dislocation was introduced into a TiN supercell. The supercell has 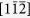 in x, 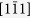 in y and 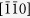 in z directions. The dislocation line direction is along z. The initial atomic structure of dislocation was created using anisotropic linear elasticity theory employing the Stroh solution. The supercell is periodic in z, and has fixed boundaries in x and y, approx. 4.1 nm and 2.9 nm respectively. To get accurate atomic forces and stresses, a 1 × 1 × 7 Monkhorst−Pack mesh for k-point sampling and a planewave kinetic energy cutoff of 500 eV for the planewave expansion of the wave functions were used in the slab calculations. A thickness of 0.6–1.0 nm vacuum is applied to the fixed boundaries to avoid boundary-boundary interaction due to the periodic nature of the planewave based DFT calculations in a supercell. The nudged elastic band method calculations were used to calculate the Peierls barrier of the partial dislocations^53^,^54^.

## Additional Information

**How to cite this article**: Li, N. *et al.* Quantification of dislocation nucleation stress in TiN through high-resolution *in situ* indentation experiments and first principles calculations. *Sci. Rep.* **5**, 15813; doi: 10.1038/srep15813 (2015).

## Supplementary Material

Supplementary Information

Supplementary Movie 1

Supplementary Movie 2

Supplementary Movie 3

## Figures and Tables

**Figure 1:**
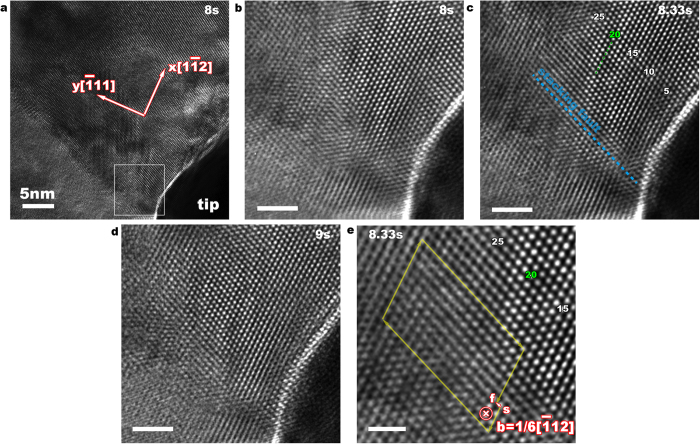
Nucleation of a partial dislocation loop in TiN under indentation along <111>. (**a**) HRTEM snapshot from *in situ* indentation. (**b)** A magnified view of the region marked with the square box in (**a**). **(c)** After 0.3 s, an offset along 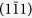 glide plane with a length of 7.5 nm, corresponding to a stacking fault, is observed. (**d)** After 1 s, The nucleated dislocation (including the trailing partial) glides into the crystal and out of the region of view. (**e)** A Burgers circuit (starting at S and ending at F) was drawn at one end by assuming the sense (direction) of the dislocation line is pointing into the plane of view. Scale bars: (**b**–**d**) 2 nm, (**e**) 1 nm.

**Figure 2:**
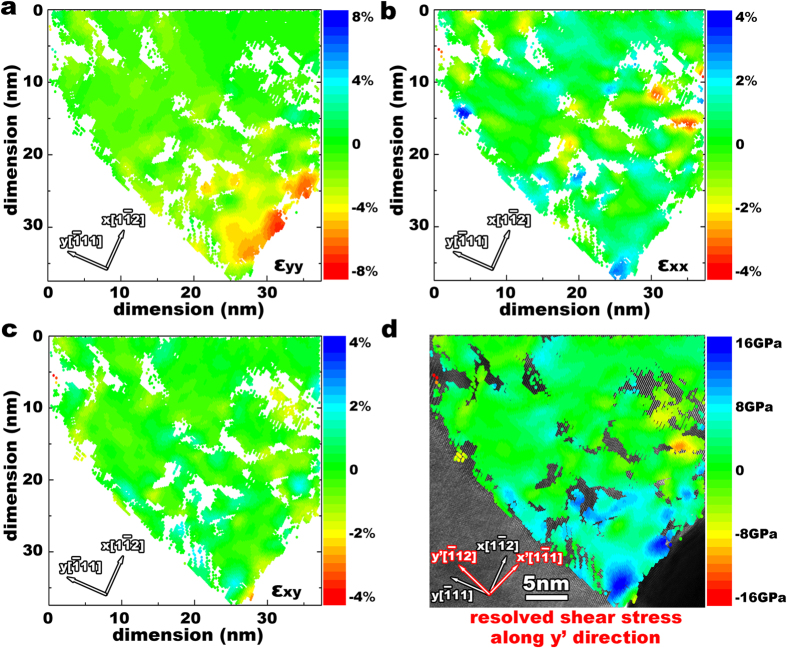
The calculated strain and resolved shear stress components in a 2D density plot. **(a)** ε_yy_; (**b)** ε_xx_; and (**c)** ε_xy_. The strain tensors are obtained in the crystal system where x = 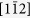 , y = 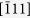 , and z = 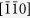 . (**d)**, The contour of the resolved shear stress on 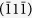 plane along y’ = 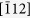 direction has been superimposed on the TEM micrograph.

**Figure 3:**
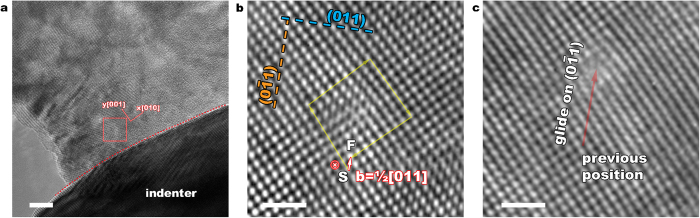
Structure evolution during the compressive loading along [100]. (**a**) Nucleation and glide of a full dislocation was observed. (**b**,**c)** HRTEM images show the trajectory of a lattice dislocation gliding on {110} plane. Scale bars: (**a**) 5 nm, (**b**,**c**) 1 nm.

**Figure 4:**
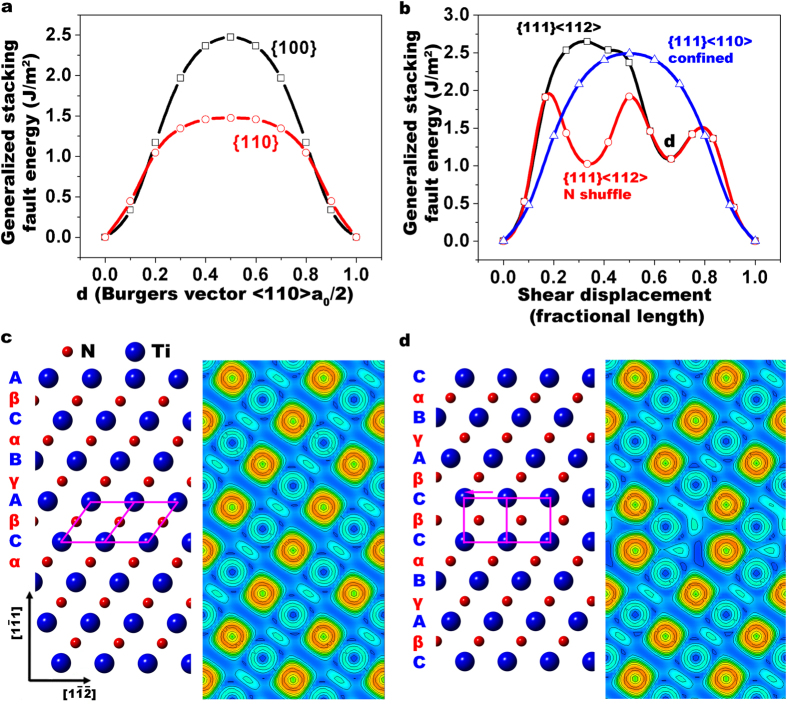
DFT calculated generalized stacking fault energies. (**a**) Generalized stacking fault energies (GSFEs) as a function of shear displacement along <110> in {110} and {100} planes. (**b**) GSFEs as a function of shear displacements along both <110> and <112> directions on {111} plane. (**c)** The atomic structures (left) and associated electron localization functions (right) of perfect TiN. (**d**) The atomic structures (left) and associated electron localization functions (right) of stacking faulted TiN.

**Table 1:** Calculated Schmid factors for uniaxial compression along <111> and <100>.

Slip systems	Indentation along <111>	Indentation along <100>
{111} <110>	0.27	0.41
{110} <110>	0	0.5
{100} <110>	0.47	0

**Table 2:** The experimental and calculated results for the two indentation directions.

	Indentation along <111>	Indentation along <100>
Dislocation nucleated	Partial a_0_/6 <211>	Full a_0_/2 <110>
Slip plane	{111}	{110}
Stacking fault width observed	7.5 nm*	Not observed
Stacking fault energy computed from DFT	1.1 J/m^2, 52^	N/A
Maximum elastic strain along indentation direction measured from HRTEM images	−7.4%	−2.6%
Local elastic stress calculated from the strain measured from HRTEM images	13.8 GPa	6.7 GPa
DFT calculated Peierls stress	2.3 GPa	1.3–1.4 GPa

^*^under current stress condition.
