# Supplementary material for: Quantification of dislocation nucleation stress in TiN through high-resolution in situ indentation experiments and first principles calculations
Source: Sci Rep. 2015 Nov 5;5:15813. doi: 10.1038/srep15813 (PMC4633591; doi:10.1038/srep15813)
Supplement: Supplementary Information [file srep15813-s1.pdf]

Quantification of dislocation nucleation stress in TiN through high-resolution *in situ* indentation experiments and first principles calculations

N. Li[1,*], S.K. Yadav[2], X.-Y. Liu[2,*], J. Wang[2,*], R.G. Hoagland[2], A. Misra[3]

* To whom correspondence should be addressed. Email: N.L. (nanli@lanl.gov), X.-Y. Liu (xyliu@lanl.gov) and J.W. (wangj6@lanl.gov).

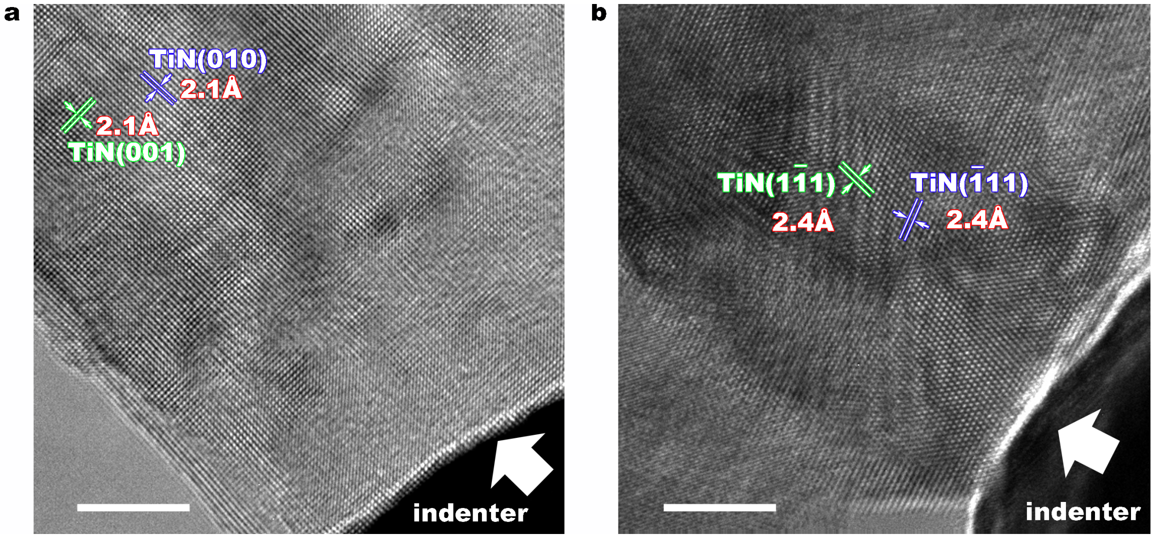

Figure S1 | XTEM micrographs of TiN films in contact with the tip of a nanoindenter inside a TEM. a, b, Indentation has been performed along two different crystallographic directions: TiN<100> and TiN<111> respectively. All scale bars, 5nm.

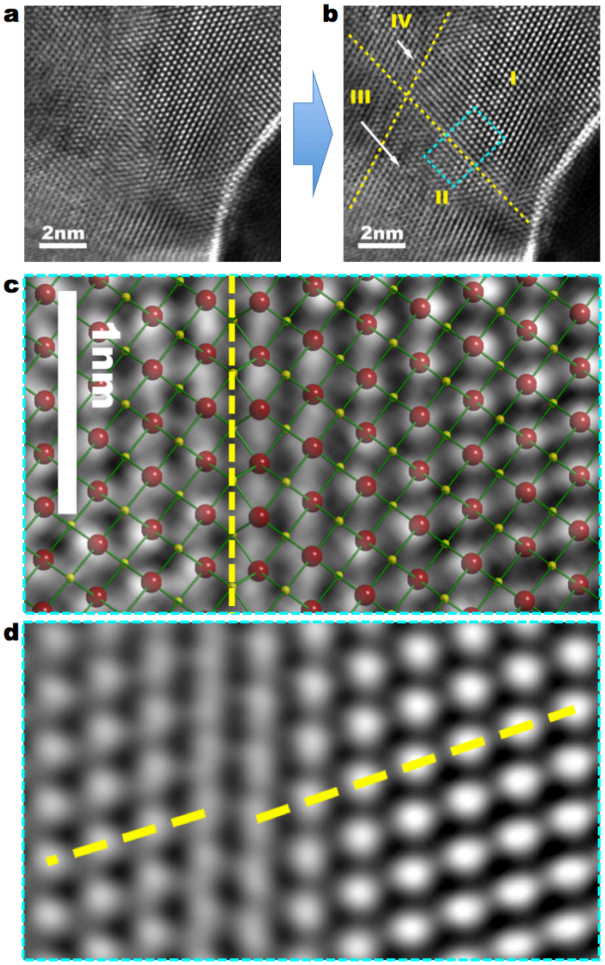

Figure S2 | The evolved structure of nucleation sites. a, b, Correlated with Supplementary Movie 2, the materials beneath the tip have been divided into four regions. The atoms in region I have been chosen as a reference and atoms in region II, III and IV shift to the lower right corner collectively, which corresponds to the nucleation of partial dislocation loop. **c, d,** Magnified image of the region in the cyan rectangle in **b** and corresponding inverse FFT image. The offset has been highlighted through overlapping schematic atomic illustration. The physical meaning of the 7.5 nm offset is that the nucleation of the full dislocation happens in two steps. In the first step, the leading partial is nucleated, and under applied stress, is able to move at least 7.5 nm before the trailing partial is nucleated. However, once the trailing partial is nucleated, it moves towards the leading partial to remove the 7.5 nm offset created due to the time lag in the nucleation of leading and trailing partials. Subsequent motion involves the full dislocation with a compact core with the 7.5 nm offset.

Method to calculate the strain/stress tensor from HRTEM images

The method does not require precise determination of the location of the atoms in the real lattice; rather it relies on measuring a change in the image relative to the unstrained image of the lattice along a low-index zone axis direction. The strain analysis is operated through two steps, (1) Calibrate the HRTEM image from an un-deformed region and (2) Calculate strains at the position of each atomic-column column with the calibrated image as a reference. The first step is to correct strains that may be generated by the distortion of the microscope.

Figures S3 illustrated how we calibrate lattice parameters from an un-deformed crystal. A HRTEM image (Fig. S3a) from the un-deformed crystal is chosen to calibrate the image distortion due to the microscope. Peak Pairs Analysis (PPA) software [1] has been applied to obtain the position for each atom-column, marked by the cyan dots. In Fig. S3b, the cyan dots have been superimposed with the ideal lattice (the red circles).

To determine the strain tensor, we used a method based on the least squares determination of the strain ellipsoid described by Hoagland, Daw and Hirth in Ref. [2]. That method, applicable to determining the 3D strain tensor, reduces, in 2D, to the least squares determination of the strain ellipse at a lattice site in the plane of a lattice image defined relative to a perfect reference lattice, and we summarize the method as follows.

Regarding the lattice image as composed of a set of points in a plane, the three local strain components in rectilinear coordinates, ε_{11}, ε_{22}, and ε_{12}, at any point surrounded by, say N, neighbors, define the radial strain in the direction of the αth neighbor, ${}_{\alpha}\varepsilon$, according to the coordinate transformation law

$${}_{\alpha}\varepsilon = {}_{\alpha} l^2 \varepsilon_{11} + {}_{\alpha} m^2 \varepsilon_{22} + {}_{\alpha} l_{\alpha} m \gamma_{12}, \qquad (1)$$

with ${}_{\alpha}l$ and ${}_{\alpha}m$ as direction cosines of the αth neighbor in a perfect (un-deformed) lattice in 2D, and higher order terms in strain have been neglected as they are assumed small. In strain space, Eq. (1) is an ellipse. Thus there are N equations of this form for each lattice point, and the left side of each equation is known since

$$ {}_{\alpha}\varepsilon = \frac{{}_{\alpha}r}{{}_{\alpha}R} - 1 \quad , \tag{2} $$

where ${}_{\alpha}r$ and ${}_{\alpha}R$ are the distances to the αth neighbor in the deformed and perfect lattices, respectively. Referring to Ref [1], the set of N equations enable the determination of the ε_{ij} components in a least squares sense giving the strains expressed as a column vector

$$ \varepsilon = \mathbf{N}^{-1}\mathbf{Q} , \tag{3} $$

where the matrix **N** and column vector **Q** are given by

$$ N = \begin{bmatrix} \sum_{\alpha} l^4 & \sum_{\alpha} l_{\alpha}^2 m^2 & \sum_{\alpha} l_{\alpha}^3 m \\ \sum_{\alpha} l_{\alpha}^2 m^2 & \sum_{\alpha} m^4 & \sum_{\alpha} l_{\alpha} m^3 \\ \sum_{\alpha} l_{\alpha}^3 m & \sum_{\alpha} l_{\alpha} m^3 & \sum_{\alpha} l_{\alpha}^2 m^2 \end{bmatrix} \text{ and } Q = [\begin{matrix} \sum_{\alpha} \varepsilon_{\alpha} l^2 \\ \sum_{\alpha} \varepsilon_{\alpha} m^2 \\ \sum_{\alpha} \varepsilon_{\alpha} l_{\alpha} m \end{matrix}] \tag{4} $$

and the sums are over all N neighbors. The least squares determination of the strain components, leading to Eq. (3), is necessitated by various uncertainties in the positions of the various lattice points extracted from the HRTEM image.

Fig. S3d-S3f presents the calculated strain components ε_{xx}, ε_{yy} and ε_{xy} for all the atoms in Fig. S3a and the strain tensors are obtained in the crystal system where x= [010], y= [001], and z= [100]. The corresponding quantitative distributions of the strain are illustrated in Fig. S3g-S3i and the mean elastic strains are -0.064%, -0.12%, and +0.47% with the standard deviation of 0.34%, 0.37%, and 0.56% respectively. The values of the mean elastic strain have been chosen as the reference. The same procedure has

been applied on atoms in the situation of straining and calculated values are calibrated in comparison to the reference to get the information of real strain.

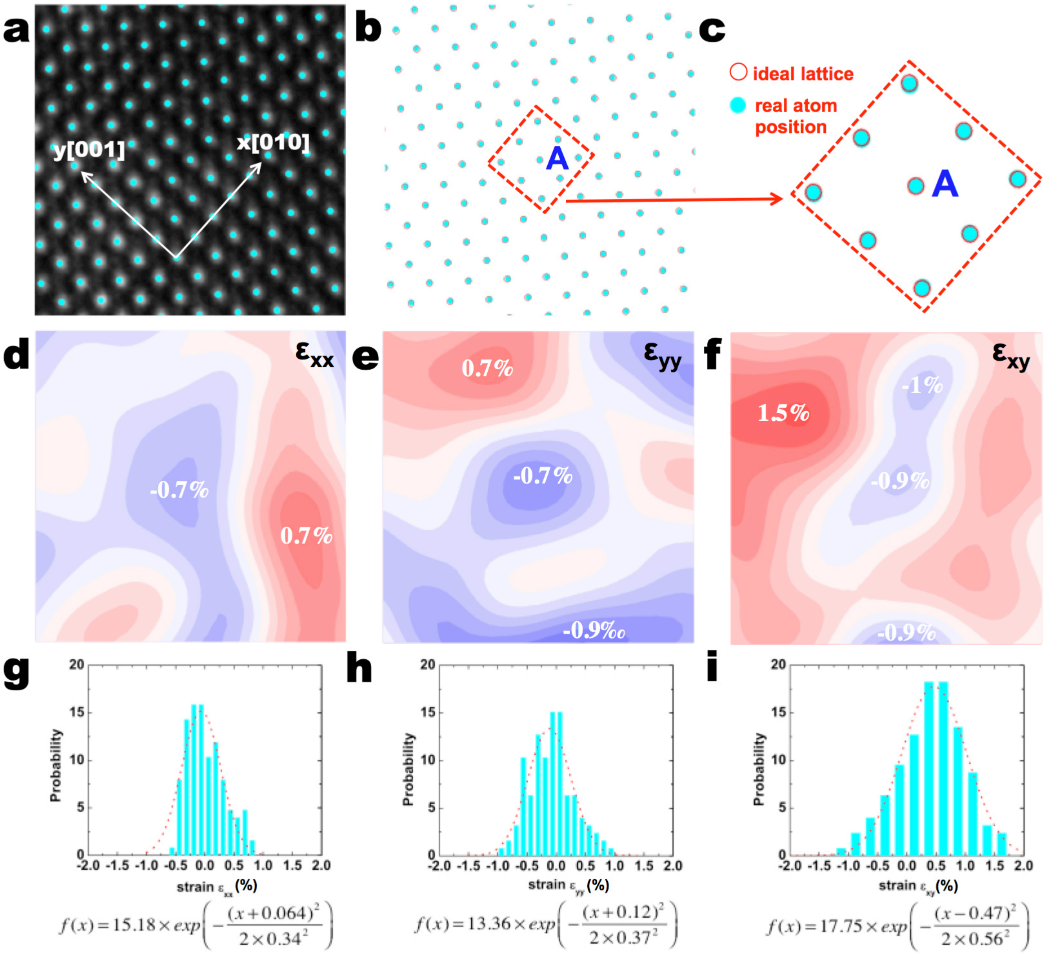

Figure S3 | Strain analysis. a, An unstrained HRTEM image from undeformed crystal with clear image of atomic columns. Overlapped cyan dots stand for the position of each atom calculated from PPA software. **b,** Superimposition of real atoms with the ideal lattice, which is in red circle. **c,** Magnified image of the region in the red dashed square in **b** to show that the first and second nearest neighbor atoms have been chosen to define the strain at A. **d-f,** The calculated strain components ε_{xx}, ε_{yy} and ε_{xy}, respectively for all the atoms in (a). The strain tensors are obtained in the crystal system where x= [010], y= [001], and z= [100]. **g-i,** Corresponding quantitative distributions of the strain components ε_{xx}, ε_{yy} and ε_{xy}.

With the determined strains, the corresponding stresses are then calculated from DFT. The DFT calculations were performed using the efficient planewave basis code

Vienna *ab initio* Simulation Package (VASP) [3,4], employing the Perdew, Burke, and Ernzerhof (PBE) [5] exchange-correlation functional and the projector-augmented wave (PAW) methodology [6]. The method for determining stresses was also checked by performing calculations on Al. Our previous DFT calculations have shown that for both Al and TiN, the calculated lattice parameters and elastic constants are in excellent agreement with experiments [7-9].

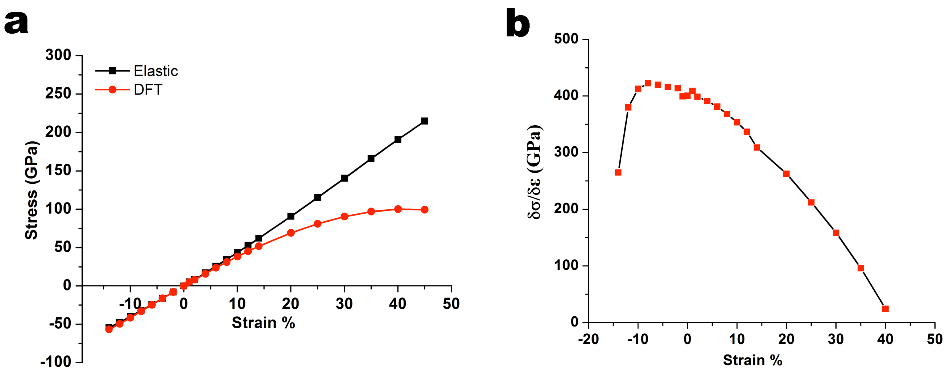

Figure S4 | Calculated stress strain relationship of TiN. a, Stress-strain curve for uniaxial stress deformation along the <111> direction of TiN. **b,** The slope of stress (*ds/de*) as a function of strain.

To understand the general trend at large strain/stress, deformations were applied to TiN, along the <111> direction. Only the uniaxial stress deformation results are shown here. We also found that DFT calculations employing Ti pseudopotentials containing 4 valence electrons ($3d^3 4s^1$) or 12 valence electrons ($3p^6 3d^4 4s^2$) do not make significant difference in the final results. The DFT calculated stress-strain curve shown in Fig. S4a is in good agreement with the linear elastic prediction at even modest high strains, from -10% to 6%. The elastic solution used the DFT strains in the two orthogonal directions, $[1\bar{1}2]$ and $[\bar{1}11]$. In Fig. S4b, the slope of stress vs. strain (*ds/de*) is shown as a function of strain. Surprisingly, in the region of 0 to 9% in compressive strain, an approximately

constant slope is obtained. We have also accommodated the possibility of internal relaxations in TiN in DFT calculations during large deformations. Random displacements were applied to N positions before the DFT structural relaxations for selected strains, -15% and 14%. For both cases, no internal relaxation between Ti and N was observed in the relaxed results.

From Fig. 2, a large strain region is identified. In the crystal system where x= $[1\bar{1}2]$, y= $[\bar{1}11]$, and z=$[\bar{1}\bar{1}0]$, the average strain tensor is $\varepsilon_{ij} = \begin{pmatrix} 0.0194 & 0.0109 \\ 0.0109 & -0.0742 \end{pmatrix}$ and the corresponding DFT stress is $\sigma_{ij} = \begin{pmatrix} -0.8 & 5.6 \\ 5.6 & -30.8 \end{pmatrix}$ (GPa). The DFT calculation was carried out with a 12 atoms TiN supercell and under a plane stress condition. The principal axes are rotated by a small angle, 6.5^0, away from $[\bar{1}11]$, where the shear strain/stress components are zero.

The slip plane of the partial dislocation emitted in a subsequent frame is $(1\bar{1}1)$, and its Burgers vector direction, $[\bar{1}12]$. The rotated crystal system is then y'=$[\bar{1}12]$, x'= $[1\bar{1}1]$, and z'=$[\bar{1}\bar{1}0]$. By transformation of the stress tensor obtained from DFT, the resolved shear stress acting on this partial dislocation provides an estimate of the critical shear stress for nucleation and emission into the crystal of 13.8 GPa. Relative to the shear modulus (*m*) of TiN, 163 GPa [10], the critical shear stress for nucleation of the partial dislocation in the *in situ* strained sample is found to be about *m*/12.

References:

[1] Galindo, P.L., Kret, S., Sánchez, A.M., Laval, J.Y. Yanez, A., Pizarro, J., Guerrero, E., Ben, T. & Molina, S.I. The peak pairs algorithm for strain mapping from HRTEM images, *Ultramicroscopy* **107**, 1186-1193 (2007).

[2] Hoagland, R.G., Daw, M.S. & Hirth, J.P. Some aspects of forces and fields in atomic models of crack tips. *J. Mater. Res.* **6(12)**, 2565-2577 (1991).

[3] Kresse, G. & Hafner, J. Abinitio molecular-dynamics for liquid-metals. *Phys. Rev. B* **47**, 558-561 (1993).

[4] Kresse, G. & Furthmüller, J. Efficiency of ab-initio total energy calculations for metals and semiconductors using a plane-wave basis set. *Comput. Mater. Sci.* **6**, 15-50 (1996).

[5] Perdew, J.P., Burke, K. & Ernzerhof M. Generalized gradient approximation made simple. *Phys. Rev. Lett.* **77**, 3865-3868 (1996).

[6] Blöchl, P.E. Projector augmented-wave method. *Phys. Rev. B* **50**, 17953-17979 (1994).

[7] Bhattacharyya, D., Liu, X.Y., Genc, A., Fraser, H.L., Hoagland, R.G. & Misra, A. Heterotwin formation during growth of nanolayered Al-TiN composites. *Appl. Phys. Lett.* **96**, 093113 (2010).

[8] Yadav, S.K., Ramprasad, R., Misra, A. & Liu, X.Y. First-principles study of shear behavior of Al, TiN, and coherent Al/TiN interfaces. *J. Appl. Phys.* **111**, 083505 (2012).

[9] Yadav, S.K., Ramprasad, R., Misra, A. & Liu, X.Y. Core structure and Peierls stress of edge and screw dislocations in TiN: A density functional theory study. *Acta Mater*. **74**, 268-277 (2014).

[10] Kim, J.O., Achenbach, J.D., Mirkarimi, P.B., Shinn, M. & Barnett, S.A. Elastic-constants of single-crystal transition-metal nitride films measured by line-focus acoustic microscopy. *J. Appl. Phys.* **72**, 1805-1811 (1992).
